# Grazing-incidence X-ray diffraction investigation of the coincidence site lattice of the Ge/Si(001) system

**DOI:** 10.1107/S1600576720009255

**Published:** 2020-08-14

**Authors:** Yvo Barnscheidt, Jan Schmidt, H. Jörg Osten

**Affiliations:** a Institute of Electronic Materials and Devices, Leibniz University Hannover, Schneiderberg 32, Hannover, 30167, Germany; b Laboratory of Nano and Quantum Engineering, Leibniz University Hannover, Schneiderberg 39, Hannover, 30167, Germany

**Keywords:** grazing incidence, X-ray diffraction, Ge/Si, heteroepitaxy, coincidence site lattices, dislocation network

## Abstract

The Ge/Si(001) system has been analysed by grazing-incidence X-ray diffraction on a standard laboratory X-ray diffraction tool. A periodic array of interfacial edge dislocations forms a coincidence site lattice (CSL) which yields equidistantly spaced satellite peaks close to Bragg peaks of the Ge layer and Si substrate. The diffraction behaviour of the CSL was analysed using 2θ/φ scans along [100], [110] and [310] directions as well as azimuthal φ scans which revealed a 90° angular symmetry of the CSL. Additionally, different layer thicknesses, from 10 to 580 nm, were analysed, focusing on the dependence of layer thickness on the glancing angles of the satellite peaks. This method provides the ability to analyse whether or not epitaxially grown layers exhibit a periodic array of dislocations, and gain information about the orientation of the interfacial edge dislocations.

## Abstract 2

Grazing-incidence X-ray diffraction analysis of the coincidence site lattice formed by interfacial misfit dislocations in Ge/Si(001) heteroepitaxy is reported for a wide variety of Ge layer thicknesses ranging from 10 to 580 nm.

## 1. Introduction   

The interface is of great importance in epitaxial growth since different materials interact directly with each other. Heteroepitaxial growth is normally accompanied by a lattice mismatch which induces mechanical strain in the film. On exceeding the critical thickness relaxation will occur, for example by the formation of misfit dislocations.

The orientation and spacing of the misfit dislocations are not always obvious and have to be analysed. Two methods are commonly used for structural analysis, transmission electron microscopy (TEM) and X-ray diffraction (XRD). The first method is capable of analysing defects and provides atomic resolution but suffers from being time consuming, expensive and destructive. The latter is perfectly suited to analysing periodic structures and has the advantages of being fast, rather cheap and nondestructive. Interfacial defects are usually analysed by TEM because of the high versatility of this technique. To overcome the limitations of TEM, a method of applying XRD to interfacial defects is needed. By applying the **0**-lattice theory (also called coincidence site lattice) of W. Bollmann (Bollmann, 1970 ▸) a lattice of dislocations can be investigated using XRD if the radiation hits the sample in grazing incidence close to the glancing angle. There are some reports in the literature about this technique for Ag/MgO(001) (Renaud *et al.*, 1998 ▸), MnAs/GaAs(001) (Satapathy *et al.*, 2005 ▸), PbSe/PbTe (Wintersberger *et al.*, 2010 ▸) and LaSrMnO/LaAlO (100) (Santiso *et al.*, 2016 ▸). However, this distinct method has scarcely been used to analyse the Ge/Si(001) system we are working with. Pre-patterned Ge/Si(001) islands (Richard *et al.*, 2011 ▸) and pre-patterned Ge/Si(001) nanopillars (Kozlowski *et al.*, 2012 ▸) have been investigated, whereas analysis of extended layers is missing. Most of the experiments found in the literature were performed at synchrotron facilities which are rather poorly available in most laboratories. In this work, we investigated several reflections of the CSL in the Ge/Si(001) system and the dependence of the layer thickness on the glancing angle. All measurements were performed on a standard laboratory XRD tool, and our samples are fully relaxed Ge layers with a wide variety of thicknesses from 10 to 580 nm grown on standard 100 mm Si(001) wafers.

## 2. Theoretical   

### 2.1. Epitaxial growth of Ge/Si(001)   

The integration of Ge in Si technology is of manifold interest (Paul, 2004 ▸; Lee *et al.*, 2017 ▸); the main challenge in achieving good crystalline Ge layers grown on Si(001) substrates is to overcome the Stranski–Krastanov growth mode which results from the 4.18% lattice mismatch. The first few monolayers will grow pseudomorphically, but after exceeding a critical thickness of three monolayers the film will relax elastically by maximizing its surface, and thus islanding occurs (Eaglesham & Cerullo, 1990 ▸). The energy balance lies between surface maximization and strain reduction. Keeping the layer smooth, a critical thickness of 11 monolayers at which plastic formation via injection of interfacial misfit dislocations will occur was predicted (Houghton, 1991 ▸) and observed (Thornton *et al.*, 1992 ▸). Using modified growth techniques, *e.g.* two-step growth (Colace *et al.*, 1998 ▸; Halbwax *et al.*, 2005 ▸; Yurasov *et al.*, 2015 ▸), graded buffering (Fitzgerald *et al.*, 1988 ▸) or surfactant-mediated epitaxy (SME) (Copel *et al.*, 1989 ▸; Wietler *et al.*, 2006 ▸), islanding can be overcome and plastic relaxation is enforced.

**Figure 1:**
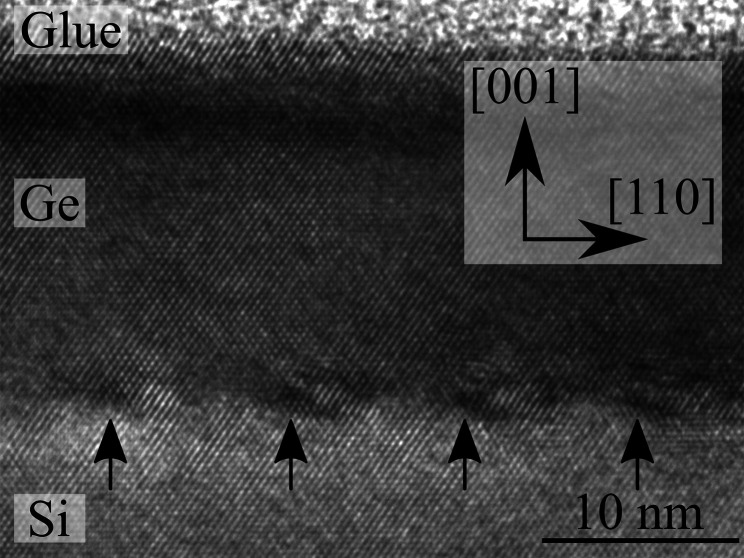
High-resolution TEM image of a 20 nm-thin, fully relaxed CME-grown Ge layer on an Si(001) substrate. The interfacial edge dislocations are marked by arrows and spaced equidistantly by 10 nm.

In plastic relaxation, the lattice mismatch is compensated by the emergence of misfit dislocations of edge type in the interface, *e.g.* a missing lattice half-plane. The edge dislocations lie in the 〈110〉 directions for (001) substrates and the length of the Burgers vector is *a*/2〈110〉 = 4 Å for *a*_Ge_ = 5.6575 Å. For a misfit of *f* = 4.18%, this gives a missing lattice half-plane every 25 lattice planes, *e.g.* a spacing of 9.6 nm (Hull & Bean, 1992 ▸). This has been observed experimentally in high-resolution (HR)-TEM (Liu *et al.*, 2012 ▸). Fig. 1 ▸ shows an HR-TEM image of a 20 nm-thin and fully relaxed Ge layer; the edge dislocations are highlighted by arrows and equally spaced by about 10 nm. Edge dislocations are terminated at the ends by 60° dislocations which penetrate to the surface of the layer. The samples investigated in this work were grown by SME or by carbon-mediated epitaxy (CME). The process flow of the SME samples was described in detail by Wietler *et al.* (2005 ▸). The CME process is described by Tetzlaff *et al.* (2012 ▸, 2013 ▸) and Barnscheidt *et al.* (2018 ▸). The Ge layers were grown on 100 mm Si(001) wafers. Both n- and p-type doped wafers were used, as well as a wide range of doping concentrations.

### 2.2. Coincidence site lattice   

**Figure 2:**
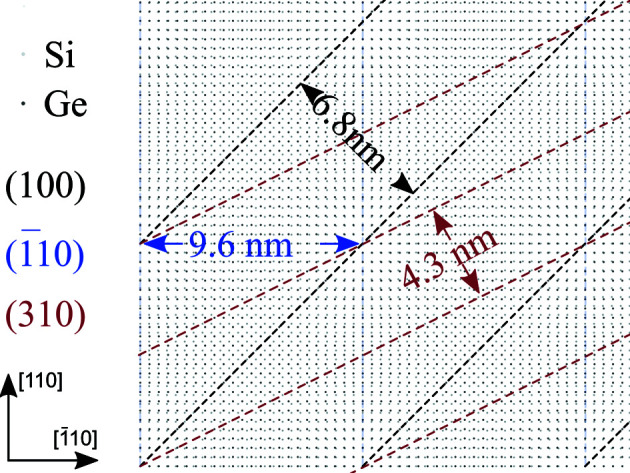
Schematic and simplified view of the Ge/Si(001) coincidence site lattice. The Si and Ge atoms are displayed as light grey and dark grey spots, respectively. The (100), 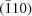 and 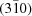 planes of the CSL are displayed in black, blue and red, respectively; the corresponding spatial distances are also shown.

The array of edge dislocations can be treated as a lattice: a ‘coincidence site lattice’ (CSL) forms (Bollmann, 1970 ▸). This lattice can be visualized by placing the Ge lattice on top of the Si lattice; a simplified sketch is displayed in Fig. 2 ▸. Light grey and dark grey spots correspond to Si and Ge atoms, respectively. The (100), (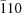) and 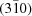 planes are displayed in black, blue and red, respectively. For each of these directions the corresponding lattice parameter of the CSL is also given. The edge dislocations are formed in the areas of poor coincidence. The CSL lattice parameter can be calculated for each crystallographic direction by dividing the corresponding film lattice parameter by the film/substrate misfit.

## 3. Experimental details   

Structural analysis was performed via TEM and XRD. An FEI Tecnai G2 F20 TMP transmission electron microscope with 200 kV acceleration voltage was used. The X-ray analysis was performed in a D8 Discover II (from Bruker AXS). A Goebel mirror was placed in front of the Cu anode. In GIXRD (grazing-incidence X-ray diffraction) setup no monochromator could be used; the excitation wavelengths are thus Cu *K*α_1_, Cu *K*α_2_ and Cu *K*β. This is important regarding the line width of the peaks and the angular splitting of the peaks for higher angles. A point detector with a scintillation counter was used. Prior to each measurement, the angle of incidence was aligned to achieve maximum intensity of the satellite peaks. A typical angle of incidence is α_c_ = 0.2°. The scan geometry was 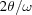; since 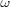 and 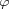 usually denote the same angles in GIXRD we will write 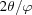 for better understanding.

**Figure 3:**
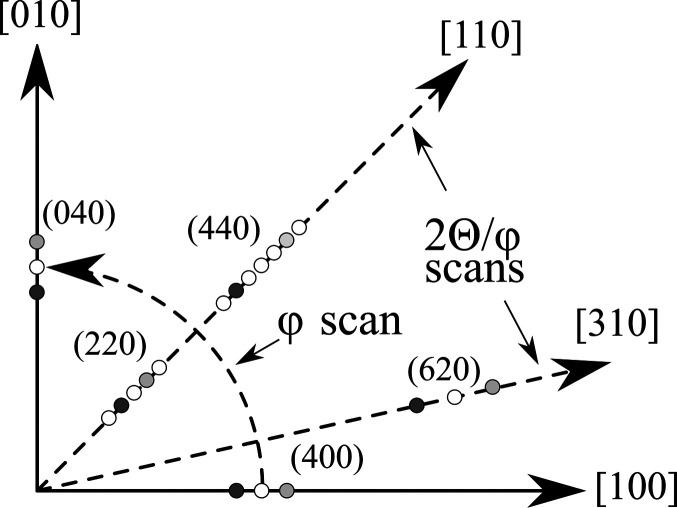
Schematic view of the reciprocal space; dark grey, light grey and white circles represent the Ge, Si and satellite reflections, respectively. Also, the scan directions of 2θ/φ and φ scans are stated.

In Fig. 3 ▸ a schematic view of the reciprocal space is displayed. Light grey and dark grey spots correspond to reflections of Si and Ge, respectively. White circles represent the occurrence of satellite peaks. We analysed the (220), (440), (400) and (620) reflections. The scan directions of 2θ/φ and φ scans are also given.

## 4. Results and discussion   

**Figure 4:**
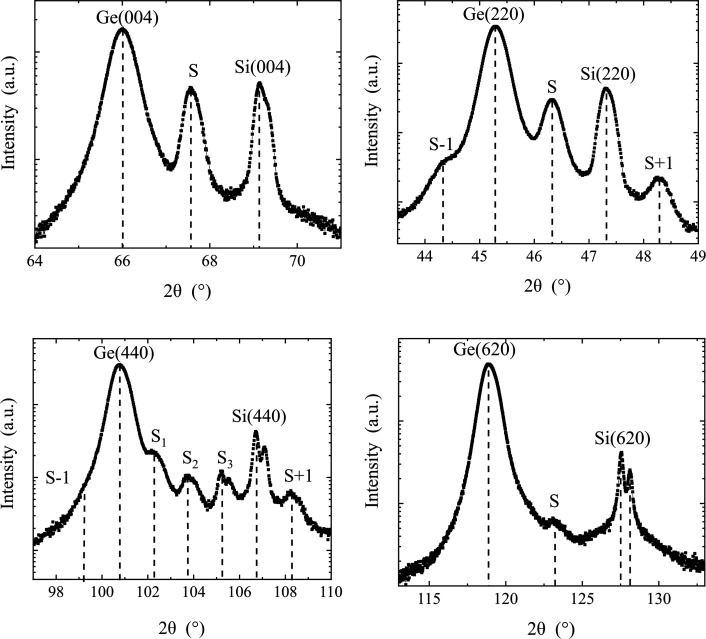
Symmetric 2θ/φ scans of the 20 nm Ge/Si(001) sample in grazing-incidence setup. The angle of incidence was aligned to achieve maximum intensity of the satellite peaks. The corresponding indices are given for each peak.

Twelve samples with Ge film thicknesses in the range from 10 to 580 nm were investigated. All samples are fully relaxed (102 < *R* < 104%) and show slight tensile strain due to the difference in thermal expansion coefficients of Si and Ge. The samples, whether grown by SME or CME, exhibit similar diffraction behaviour caused by dislocations. For our analysis, we focus on the measurements of a sample with a 20 nm Ge layer grown on an Si(001) substrate. Measurements on four different reflections were performed. The 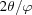 scans are depicted in Fig. 4 ▸, and the corresponding (400), (220), (440) and (620) positions are given for each peak. We remind readers that Cu *K*α_1_ and Cu *K*α_2_ wavelengths are present, which results in a doublet peak for each reflection. This is particularly visible at higher angles as seen in the (440) and (620) diffractograms in Fig. 4 ▸. The reflections are indicated by dashed lines in each diagram and denoted with Ge, Si and S. Here, S stands for satellite; S−1,2,… and S+1,2,… denote satellites at the lower-angle side of Ge and the higher-angle side of Si, respectively. Also, a wider range was measured but the satellite peaks only appear close to Bragg peaks.

The peaks appear equidistantly spaced as predicted by theory. Gaussian fit functions were used to analyse the peaks and the results are summarized in Table 1 ▸. For the (004) and the (620) scans, only one satellite peak was observed, which is due to the low lattice-plane density and high lattice-plane index, respectively. Here, the Ge–S and S–Si distances were used. The beam path of GIXRD on the CSL is comparable to that of the standard X-ray reflectometry technique; thus we can apply the well known equation (1) (Blanton & Hoople, 2002 ▸), which is usually used to analyse thickness fringes: 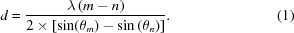
Here, λ is the X-ray wavelength, *m* and *n* are the peak indices, and *d* is the periodic distance in the CSL. These results are also summarized in Table 1 ▸. In Fig. 2 ▸ (Section 2.2), we can see a good agreement with the values we calculated from our measurements (see Table 1 ▸).

**Figure 5:**
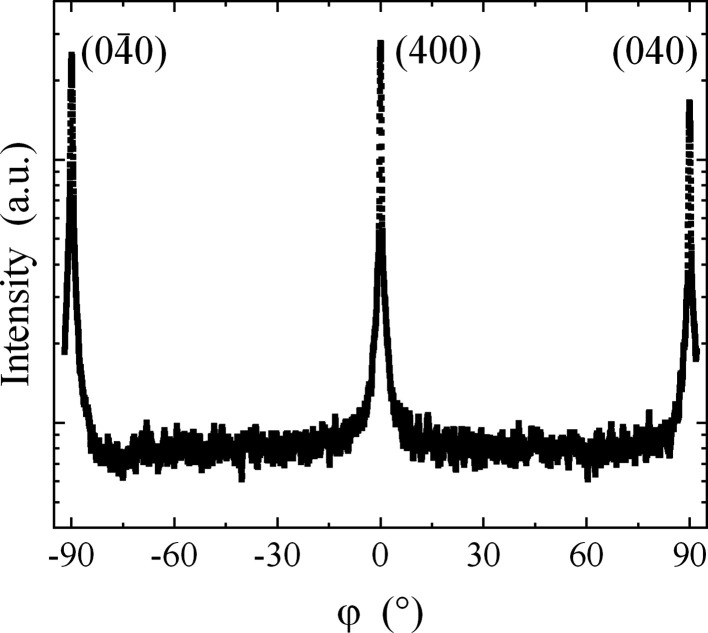
φ scan (±92°) of the satellite S at 2θ = 67.6°, as displayed in Fig. 3 ▸. A 90° periodicity is observed.

We also performed ‘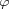 scans’, where the angle of the detector was constant and the sample was rotated around the surface normal, *i.e.* an azimuthal scan in reciprocal space (see Fig. 2 ▸). A typical 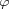 scan of the (400) satellite at 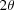 = 67.7° is shown in Fig. 5 ▸. This was also performed for the (220) reflection (not shown here) and both scans reveal a 90° symmetry, as expected by the CSL theory.

**Figure 6:**
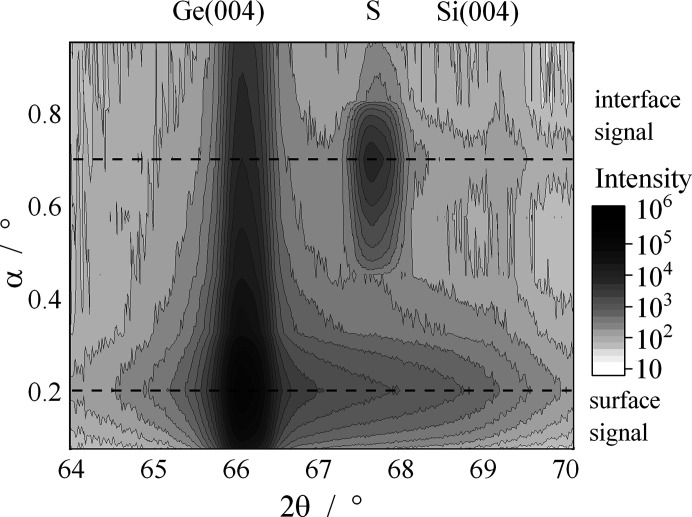
Multiple 2θ/φ scans of a 390 nm-thick Ge film, taken at variable angles of incidence 0.08 < α_i_ < 0.95°.

**Figure 7:**
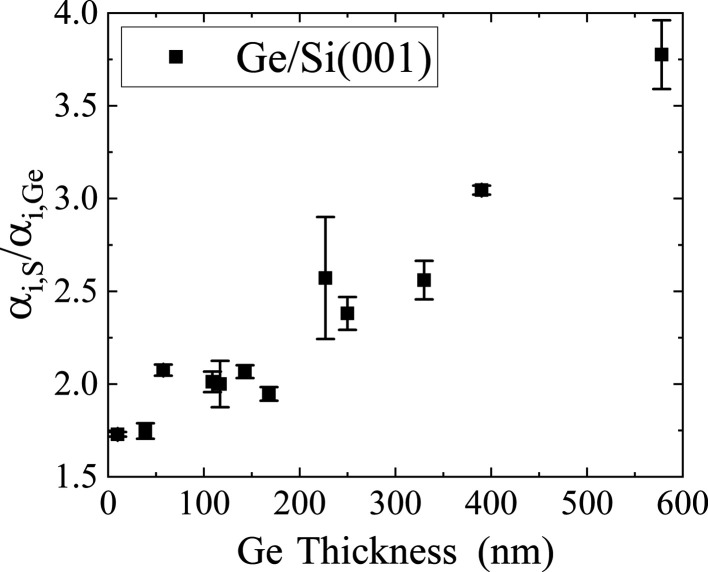
Thickness dependence of the ratio of the critical angles of incidence α_i_ of the satellite S and the Ge peak.

For a rather thick sample of 390 nm Ge grown on Si(001) substrate, we performed 2θ/φ scans of the (004) reflection with variation of the angle of incidence 0.08 < α_i_ < 0.95°; the result is displayed in Fig. 6 ▸. The higher angle of incidence gives a higher depth of penetration of the X-rays into the sample. The Ge(004) reflection at 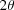 = 66.06° is visible for the full range of 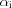. For the maximum intensity of Ge(004) at 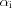
= 0.22°, a broadening to the higher-angle side is observed, which is possibly due to dynamical scattering of X-rays (Bernhard *et al.*, 1987 ▸). At 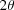 = 69.13° the Si(004) reflection can be observed for higher 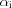 values. This means that X-rays penetrate to the substrate and can interfere constructively for Ge top layers as thick as 390 nm. Exactly in between the Ge(004) and Si(004) reflections, the additional satellite peak S is located at 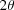 = 67.6°. This peak is visible for 0.45 < α_i_ < 0.92°. The maximum intensity of the S peak is observed at the same 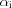 value as the maximum of the Si(004) peak. We would have expected more intensity for the Si peak than for the satellite, and it is not clear to us why the opposite was observed in Fig. 6 ▸. However, these scans give the information of an interfacial pattern. In the work of Renaud *et al.* (1998 ▸) the authors observed that the maximum intensity of these kinds of satellites is seen for 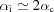, where 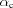 is the glancing angle. The maximum layer thickness in the work of Renaud *et al.* (1998 ▸) was 130 nm. For our 390 nm layer, the maximum intensity of the satellite was observed for 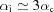. To investigate this correlation, we performed a series of 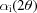 scans. We aligned the setup on the Ge and S reflections, varied 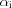 and measured the maximum intensity. This corresponds to a scan parallel to the ordinate in Fig. 6 ▸. The results are displayed in Fig. 7 ▸. The error of the measurement is based on the finite step size of our X-ray source (angle of incidence) and on the limited precision of the fitting procedure. The ratio 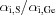 is around 1.75 for layers thinner than 50 nm. For layers with thicknesses 50 < 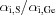 < 200 nm we get a value of around 2. For thicker layers, the ratio increases and does not saturate up to a thickness of 580 nm.

## 5. Conclusion   

Fully relaxed Ge layers with thicknesses ranging from 10 nm up to 580 nm were grown on Si(001) substrates and analysed by TEM and (GI)XRD. The misfit dislocations are equi­distantly spaced and form a coincidence site lattice. Using the glancing angle of the layer material in GIXRD, only the layer peak can be observed. When increasing the angle of incidence to >1.75α_c_, depending on substrate thickness, the substrate reflection appears as well as additional satellite peaks close to Bragg peaks. The maximum intensity of the satellite peaks arises for the same 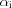 as for the Si substrate reflection. This clearly shows that the satellite peaks result from an interfacial pattern. The satellite peaks were investigated for 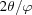 scans of the (400), (220), (440) and (620) reflections. For all these scans, equidistantly spaced satellite peaks were observed. The distance of the CSL planes corresponds to the angular distance of the equidistantly spaced satellite peaks in each direction according to the equation of Blanton and Hoople which is usually used for thickness fringe analysis. Azimuthal scans reveal a 90° symmetry of the satellite peaks, which is in accordance with the CSL model and the 〈110〉 orientation of the dislocations. A graphical scheme of the CSL was shown, including the (100), 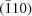 and 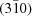 CSL lattice planes. This scheme is in agreement with the diffractogram analysis using the equation of Blanton & Hoople (2002 ▸).

The ratio of the glancing angles of the satellite peaks S and the glancing angle of the Ge layer, 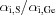, was investigated for layer thicknesses ranging from 10 to 580 nm. The ratio is around a value of 2 for layer thicknesses from 50 to 200 nm. For thicker layers, the ratio increases linearly to around 3.8 for the thickest layer of 580 nm.

We used a standard laboratory XRD tool to analyse the spacing and orientation of misfit dislocations in Ge/Si(001) epitaxy with a GIXRD setup. This method is a fast way to investigate the interfacial dislocations pattern of heterostructures.

## Figures and Tables

**Table 1:** Angular distance Δ of the equidistantly spaced peaks of the 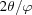 scans and the calculated lattice parameter *d* for each crystallographic direction

	(400)	(220)	(440)	(620)
Δ (°)	1.5609	0.9973	1.506	4.3203
*d* (nm)	6.8	9.63	9.62	4.3
